# Predictors of in-hospital mortality in traumatic subarachnoid hemorrhage: a nationwide study of 67,684 patients in Germany

**DOI:** 10.1016/j.bas.2026.106049

**Published:** 2026-04-15

**Authors:** Jan Reinhard, Melanie Ardelt, Josina Straub, Jonas Krückel, Amer Haj, Markus Rupp, Nils Ole Schmidt, Volker Alt, Tobias Renkawitz, Siegmund Lang

**Affiliations:** a Department of Orthopaedics, Regensburg University, Kaiser-Karl V. Allee 3, 93077, Bad Abbach, Germany; b Department of Trauma Surgery, University Medical Center Regensburg, Franz-Josef-Strauß-Allee 11, 93053, Regensburg, Germany; c Division of Orthopaedics and Traumatology, University Hospital Krems, Krems, Austria; d University for Continuing Education, Danube University Krems, Krems, Austria; e Department of Trauma, Hand- and Reconstructive Surgery, University Hospital Giessen, Giessen, Germany; f Department of Neurosurgery, University Medical Center Regensburg, Regensburg, Germany

**Keywords:** Traumatic subarachnoid hemorrhage (tSAH), In-hospital mortality, Epidemiology, Complications, Incidence rate, Nationwide registry (InEK), Geriatric patients

## Abstract

**Introduction:**

Population-based data on incidence and mortality risk factors, particularly in very elderly patients with traumatic subarachnoid hemorrhage (tSAH) are limited. This study analyzed epidemiology, comorbidities, complications, age-specific incidence rates, and predictors of in-hospital mortality in patients with tSAH in Germany, with a focus on those aged ≥80 years.

**Material and methods:**

This retrospective cross-sectional study included all hospitalized patients with tSAH (ICD-10 S06.6) in Germany from 2019 to 2022, using nationwide administrative data from the Institute for the Hospital Remuneration System (InEK-GmbH). Demographics, comorbidities, complications, diagnostic procedures, ICU treatment, and in-hospital mortality were analyzed. Odds ratios (ORs) with 95% confidence intervals (CIs) were calculated using univariable analyses. Age-specific incidence rates were derived from Federal Statistical Office (Destatis) population data.

**Results:**

In a nationwide cohort of 67,684 tSAH patients, in-hospital mortality was 9.1%. The incidence of tSAH was 34.4 per 100,000 and increased sharply with age. Hypertension (44.5%) and atrial fibrillation (14.8%) were common. Mortality was higher in patients with atrial fibrillation (OR 1.86), cardiac pacemakers (OR 1.65), and three-vessel coronary artery disease (OR 1.54). Prolonged unconsciousness (>24 h) was the strongest predictor of death (OR 16.32), followed by cerebral edema (OR 4.70). Septic shock, renal failure, and traumatic shock carried the highest mortality risk.

**Discussion and conclusion:**

tSAH is associated with substantial in-hospital mortality, particularly in very elderly patients and those with cardiac comorbidities. These predictors/associations may support risk stratification and early intensive care management.

## Highlights

• In a nationwide cohort of 67,684 tSAH patients, in-hospital mortality was 9.1%.
• The incidence of tSAH was 34.4 per 100,000 and increased sharply with age.
• Key predictors: AF, pacemaker, 3-vessel CAD; anticoagulants slightly ↑ risk.
• Strongest risks: coma >24 h; septic shock, renal failure, traumatic shock.

## 1 Introduction

Subarachnoid hemorrhage (SAH) demonstrates a neurological emergency with reported mortality rates of up to 50% (Long et al., 2017; Griswold et al., 2022). Traumatic subarachnoid hemorrhage (tSAH) following traumatic brain injury (TBI) represents the most common cause (Long et al., 2017). Falls, traffic accidents and violence are the main reasons for TBI (Griswold et al., 2022). The incidence of TBI is reported to be threefold higher in low-and middle-income countries in comparison to high-income countries (Dewan et al., 2019; Tropeano et al., 2019). However, global incidence rates of TBI have decreased significantly since 1990 (Guan et al., 2023; León-Carrión et al., 2005). Contemporary data reveal important shifts in the demographics of tSAH/TBI patients, especially regarding age distribution (Steudel et al., 2005). Once considered primarily a problem of young adult males (due to traffic accidents and high-energy trauma), TBI epidemiology in high-income countries has progressively shifted towards older age groups in recent years (Steudel et al., 2005). In Germany, a recent multicenter study observed bimodal incidence peaks: one in early adulthood (predominantly in young men) and another in the elderly, with a notable surge in patients ≥75 years (Schwenkreis et al., 2021). This trend is driven largely by falls in the elderly, which have overtaken road traffic collisions as the leading cause of TBI in many high-income countries. In this elderly population, prone to falls comorbidities such as atrial fibrillation, oral anticoagulant use, renal failure, and cardiac disease are frequent, all of which are suspected to increase in-hospital mortality, which we aimed to quantify in a nationwide cohort (Steyerberg et al., 2019; Rhodes et al., 2023). Further, we hypothesize that concomitant injuries including skull fractures, cerebral edema, and prolonged unconsciousness can be associated with worsened outcomes, often reflecting severe traumatic mechanisms (Tiruneh et al., 2020).

TSAH warrants separate investigation because it represents a distinct subtype of TBI with unique pathophysiological mechanisms, clinical trajectories, and prognostic implications that are not fully captured when analyzed within aggregated TBI cohorts. Despite tSAH's clinical significance, there is a notable scarcity of large, nationwide studies examining its epidemiology and outcomes. This lack of robust data is problematic, as it hinders our ability to identify macro-level trends and risk factors that could inform public health interventions (Younsi et al., 2023). Real-world population-based analyses are needed to validate findings from smaller studies and to provide external benchmarks for clinical outcomes.

In this context, we set out to leverage a large national hospital discharge dataset from Germany to gain contemporary insights into tSAH. The analysis is based on ICD-10-coded administrative data and does not include clinical severity measures such as the Glasgow Coma Scale. Specifically, the present study aims to characterize the incidence of tSAH, patient demographics, associated comorbidities and injuries, and predictors of in-hospital mortality on a nationwide scale.

### 1.1 Purpose of the study

This study had four aims: first, to characterize demographics, comorbidities and complications in all hospitalized tSAH patients; second, to calculate age-specific incidence rates in Germany; third, to identify risk factors for in-hospital mortality related to secondary diagnoses, diagnostic procedures and complications; and fourth, to conduct a dedicated analysis of patients aged ≥80 years.

## 2 Methods

In a retrospective, cross-sectional nationwide, hospital discharge-based study design, all patients having tSAH (ICD-10 code: S06.6) with need for in-hospital treatment between 2019 and 2022 in Germany were identified. Data was imposed by the Institute for the Hospital Remuneration System in Germany (InEK GmbH, Siegburg, Germany). The InEK database contains mandatory hospital discharge data from all acute care hospitals in Germany, including ICD-10 diagnoses, procedures and discharge status. Coding accuracy is incentivised by the DRG-based reimbursement system, although miscoding cannot be fully excluded. Data was gathered, using the InEK data browser (https://datenbrowser.inek.org, accessed in 01/07/2023). All provided data is anonymized. All patients suffering from tSAH (ICD-10 code: S06.6) as primary diagnosis were enrolled (https://www.bfarm.de/EN/Code-systems/Classifications/ICD/ICD-10-GM/_node.html). Multiple admissions of the same patient (transfers, readmissions) could not be excluded. Total case numbers, demographic data (sex, age), secondary diagnosis, comorbidities, need for intensive care therapy, use of diagnostic instruments, complications, performed procedures and the number of in-hospital deaths were imposed. Age-specific incidence rates were calculated per calendar year and averaged afterwards. The primary endpoint was all-cause in-hospital mortality during the index admission. As we assumed increased in-hospital mortality in patients above 80 years due to tSAH from falling and existing comorbidities such as atrial fibrillation, oral anticoagulant use and renal failure, this cohort was analyzed separately. The Odds ratio (OR) and confidence interval (CI) for in-hospital mortality were calculated considering secondary diagnosis, diagnostic procedures, and complications as potential risk factors.

The overall incidence of tSAH was calculated based on population figures from 2019 to 2022. To determine age-specific incidence rates in Germany for the years 2019 to 2022, we used population data from Destatis (Federal Statistical Office of Germany) in conjunction with reported case numbers of tSAH across different age groups (https://www.destatis.de/DE/Home/_inhalt.html, accessed 01/05/2025). Incidence rates per 100,000 individuals were calculated using the formula: Incidence = (Number of cases/Total population of the age group) × 100,000.

According to national regulations, formal ethics approval was not required for analyses of anonymized administrative data.

### 2.1 Statistical analysis

Data were summarized using absolute and relative frequencies. Univariable analyses were performed to assess associations between categorical variables and in-hospital mortality using Chi-square tests. Univariable odds ratios (OR) with corresponding 95% confidence intervals (95% CI) were calculated. Statistical significance was defined as a two-sided p-value ≤0.05. Due to limitations of the available dataset with lack of individual-level linkage, multivariable modelling was not feasible. Given the observational nature of the study, the identified variables should be interpreted as factors associated with in-hospital mortality rather than causal predictors.

All statistical analyses were performed using IBM SPSS Statistics 28 (International Business Machines Corporation, Armonk, NY, USA) and Microsoft Excel 2019 (Microsoft Corporation, Redmond, WA, USA).

## 3 Results

### 3.1 Baseline analysis

Between 2019 and 2022, 67,684 patients in Germany were hospitalized with primary diagnosis of tSAH (ICD-10 S06.6). In-hospital mortality was 9.1% (n = 6166). The cohort included 37,318 males and 30,366 females, with male sex slightly increasing mortality risk (OR 1.14). Mean hospital stay was 10.2 ± 15.4 days, and 52.1% required intensive care.

**Fig. 1:**
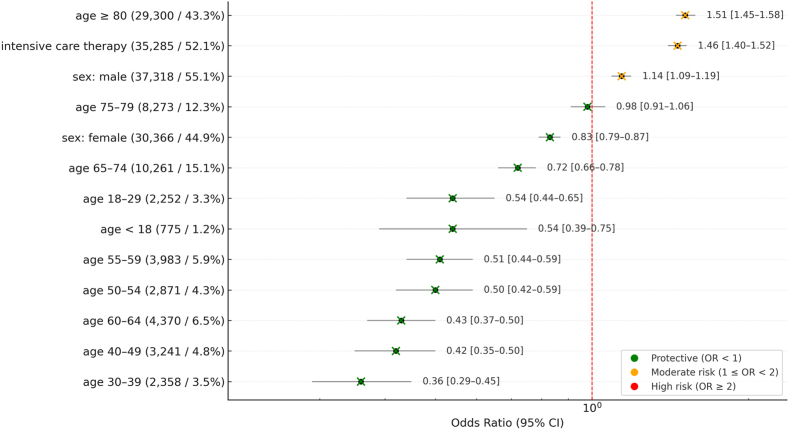
Forrest Plot of demographic data providing Odds ratio (OR) and 95% confidence interval (CI) for in-hospital mortality of the 67,684 included patients with traumatic subarachnoid hemorrhage (tSAH).

Notably, 43.3% of patients were ≥80 years, prompting separate subgroup analysis. The overall annual incidence of tSAH in Germany was 34.4 per 100,000 (Table 1, Fig. 1). Age-specific incidence increased progressively with age, from 5.8 per 100,000 population in individuals aged <60 years to 31.3 per 100,000 in those aged 60–79 years, and reaching 123.4 per 100,000 in patients aged ≥80 years, representing the highest burden across all age groups.

**Table 1:** Demographic data of the 67,684 patients with traumatic subarachnoid hemorrhage (tSAH). Patients’ age is noted in periods of ten years or lower 18 or above 80 years. Absolute and relative frequencies for age, sex and need for intensive care therapy is noted as well as Odds ratio (OR), 95% confidence interval (CI) and p-value for in-hospital mortality.

		absolute (relative) frequencies	Deaths (absolute frequencies)	OR (95%-CI) for in-hospital mortality	p-value (Chi-Squared)
age (years)	<18	775 (1.2%)	40	0.54 (0.39 – 0.75)	<0.001
	18-29	2252 (3.3%)	115	0.54 (0.44 – 0.65)	<0.001
	30-39	2358 (3.5%)	83	0.36 (0.29 – 0.45)	<0.001
	40-49	3241 (4.8%)	130	0.42 (0.35 – 0.5)	<0.001
	50-54	2871 (4.3%)	137	0.5 (0.42 – 0.59)	<0.001
	55-59	3983 (5.9%)	193	0.51 (0.44 – 0.59)	<0.001
	60-64	4370 (6.5%)	181	0.43 (0.37 – 0.5)	<0.001
	65-74	10,261 (15.1%)	689	0.72 (0.66 – 0.78)	<0.001
	75-79	8273 (12.3%)	741	0.98 (0.91 – 1.06)	0.97
	>80	29,300 (43.3%)	3857	1.51 (1.45 – 1.58)	<0.001
sex (male/total)		37,318/67,684	3834	1.14 (1.09 – 1.19)	<0.001
sex (female/total)		30,366/67,684	2332	0.83 (0.79 – 0.87)	<0.001
hospitalization (days) (mean ± SD)			10.2 ± 15.4		
intensive care therapy		35,285 (52.1%)/67,684	4505	1.46 (1.4 – 1.52)	<0.001
deaths		6166 (9,1%)/67,684			
overall incidence Germany		34.4/100,000

### 3.2 Risk factors for in-hospital mortality in terms of secondary diagnosis

**Fig. 2:**
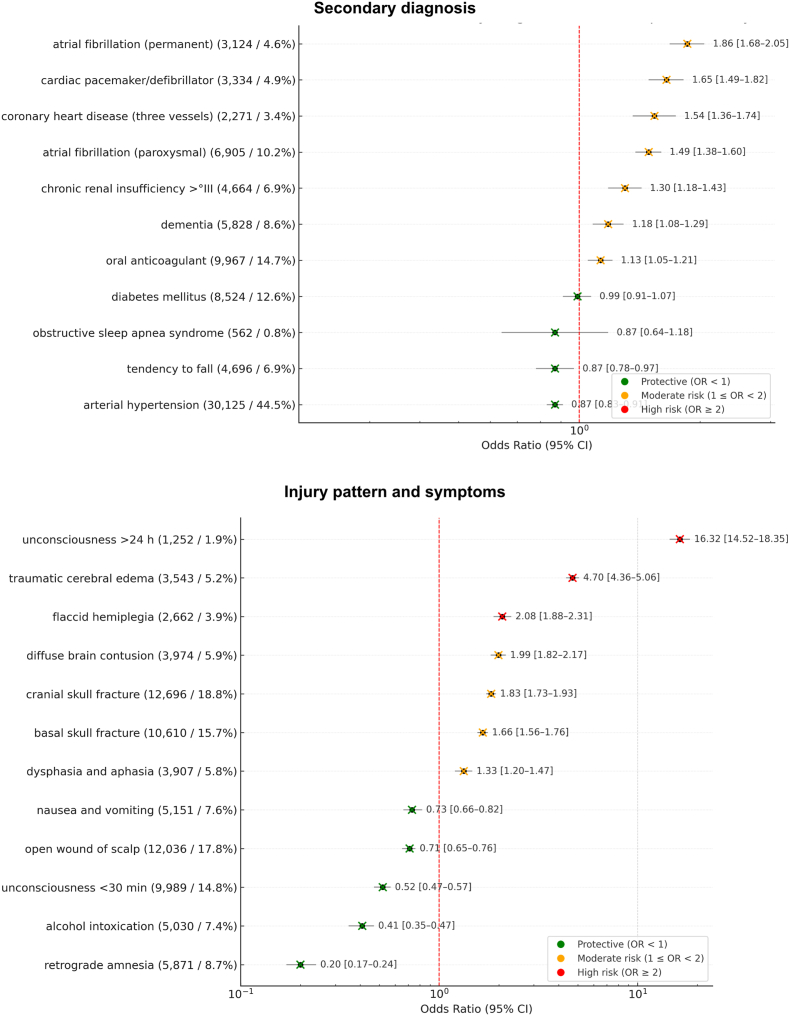
Forrest Plot of secondary diagnosis as well as injury pattern and symptoms. Providing absolute and relative frequencies, Odds ratio (OR) and 95% confidence interval (CI) for in-hospital mortality of the 67,684 included patients with traumatic subarachnoid hemorrhage (tSAH).

The most common comorbidities were hypertension (44.5%), oral anticoagulant use (14.7%), atrial fibrillation (14.8%), diabetes (12.6%), dementia (8.6%), tendency to fall (6.9%), and chronic renal insufficiency (6.9%). Cardiac conditions showed the strongest association to increase in-hospital mortality: permanent atrial fibrillation (490 deaths/3124 diagnoses, OR 1.86), cardiac pacemaker (472 deaths/3334 diagnoses, OR 1.65), and three-vessel coronary artery disease (303 deaths/2271 diagnoses, OR 1.54). Oral anticoagulants were linked to a modest increase (1013 deaths/9967 diagnoses, OR 1.13). (Fig. 2).

### 3.3 Risk factors for in-hospital mortality in terms of injury pattern and symptoms

Frequent injuries included cranial skull fractures (18.8%), scalp wounds (18.8%), basal skull fractures (15.7%), and brief unconsciousness (<30 min, 14.8%). The strongest association to increased mortality were unconsciousness >24 h (777 deaths/1252 diagnoses, OR 16.32), traumatic cerebral edema (1134 deaths/3543 diagnoses, OR 4.70), flaccid hemiplegia (460 deaths/2662 diagnoses, OR 2.08), and skull fracture (1966 deaths/12,696 diagnoses, OR 1.83). In contrast, short unconsciousness was protective (492 deaths/9989 diagnoses, OR 0.52), as was alcohol intoxication (198 deaths/5030 diagnoses, OR 0.41) (Fig. 2).

### 3.4 Frequency of used diagnostic instruments and performed procedures

Non-contrast cranial CT was the primary diagnostic tool (92.8%), followed by spinal CT (42.1%), while MRI was less common (7.4% native, 3.2% contrast) and angiography rare (1.8%). The most frequent procedures were head suturing (13.5%) and endotracheal intubation (6.7%); burr hole trepanation (4.2%), ICP monitoring (2.8%), and ventricular drainage (2.1%) were less common, and major neurosurgical interventions each occurred in <2%. The low rate of angiography use may reflect the predominance of trauma-related cases and a consequently lower clinical suspicion for aneurysmal subarachnoid hemorrhage. (Table 2).

**Table 2:** Absolute and relative frequencies of the use of diagnostic tools and procedures in the 67,684 patients with tSAH, demonstrating the most frequent ones. CT = Computed tomography. MRI = Magnetic resonance imaging.

diagnostic imaging tools	absolute (relative) frequencies
CT of head (without contrast agent)	62,835 (92.8%)
CT of spine (without contrast agent)	28,519 (42.1%)
CT of head (with contrast agent)	10,543 (15.6%)
CT of neck (with contrast agent)	8980 (13.3%)
MRI of head (without contrast agent)	4992 (7.4%)
MRI of head (with contrast agent)	2172 (3.2%)
angiography head	1206 (1.8%)


**procedures**	**absolute (relative) frequencies**

primary suture head	9111 (13.5%)
endotracheal intubation	4545 (6.7%)
burr hole trepanation	2832 (4.2%)
implantation intracranial pressure measurement	1874 (2.8%)
implantation ventricular liquor drainage	1431 (2.1%)
trepanation skull	1017 (1.5%)
duraplasty	907 (1.3%)
craniotomy	730 (1,1%)
decompressive craniectomy	695 (1%)

### 3.5 Risk factors for in-hospital mortality in terms of complications

**Fig. 3:**
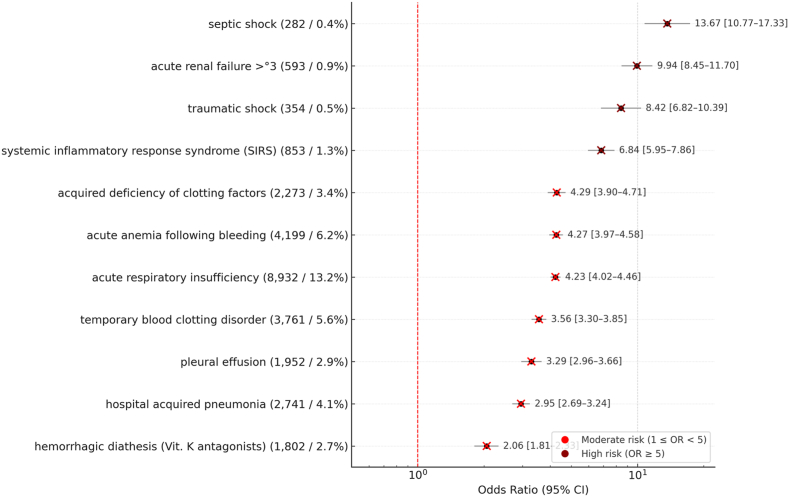
Forrest Plot of complications with absolute and relative frequencies providing Odds ratio (OR) and 95% confidence interval (CI) for in-hospital mortality of the 67,684 included patients with traumatic subarachnoid hemorrhage (tSAH).

Complications included respiratory insufficiency (13.2%), anemia (6.2%), and clotting disorders (5.6%). Mortality was most strongly associated with septic shock (163 deaths/282 diagnoses, OR 13.67), acute renal failure >°3 (296 deaths/593 diagnoses, OR 9.94), and traumatic shock (162 deaths/354 diagnoses, OR 8.42), with additional risks from clotting factor deficiency, respiratory failure, and anticoagulant-related coagulopathy (Fig. 3). These variables likely represent downstream consequences of severe injury rather than independent determinants.

### 3.6 Subgroup analyses of geriatric patients, aged above 80 years

Of all patients with tSAH, 43.3% (n = 29,398) were aged ≥80 years. Compared with younger patients, this group had higher in-hospital mortality (3857 deaths/29,398 diagnoses, OR 1.51). Within the elderly subgroup, ICU treatment (2584 deaths/13,394 diagnoses, OR 2.38) and male sex (2200 deaths/12,843 diagnoses, OR 2.06) were strongly associated with higher in-hospital mortality. All reported associations are unadjusted and should be interpreted with caution, as they are likely confounded by age and overall frailty.

### 3.7 Cardiovascular comorbidities in patients aged above 80 years

The most common cardiovascular conditions included oral anticoagulant use (23%), atrial fibrillation (paroxysmal 16%, permanent 8.3%), pacemaker/defibrillator (8.2%), and three-vessel coronary artery disease (4.8%). The strongest associations with in-hospital mortality were observed for permanent atrial fibrillation (411 deaths/2421 diagnoses, OR 2.04), pacemaker/defibrillator (389 deaths/2390 diagnoses, OR 1.94), and three-vessel coronary artery disease (223 deaths/1399 diagnoses, OR 1.89).

### 3.8 Neurological findings in patients aged above 80 years

Unconsciousness <30 min (10.8%) was most frequent, whereas unconsciousness >24 h was rare (1.3%) but showed the strongest association with mortality (320 deaths/386 diagnoses, OR 48.37). Flaccid hemiplegia (324 deaths/1079 diagnoses, OR 4.28) and dysphasia/aphasia (318 deaths/1678 diagnoses, OR 2.33) were also strongly associated with death.

### 3.9 Infectious and respiratory complications in patients aged above 80 years

Urinary tract infection (11.5%) and respiratory insufficiency (11.1%) were the most common complications. The variables most strongly associated with in-hospital mortality were septic shock (68 deaths/85 diagnoses, OR 39.91), traumatic shock (44 deaths/70 diagnoses, OR 16.88), and respiratory complications (1408 deaths/3246 diagnoses, OR 7.64 for acute respiratory insufficiency; 354 deaths/1069 diagnoses, OR 4.94 for hospital acquired pneumonia).

### 3.10 Renal and haematological complications in patients aged above 80 years

**Fig. 4:**
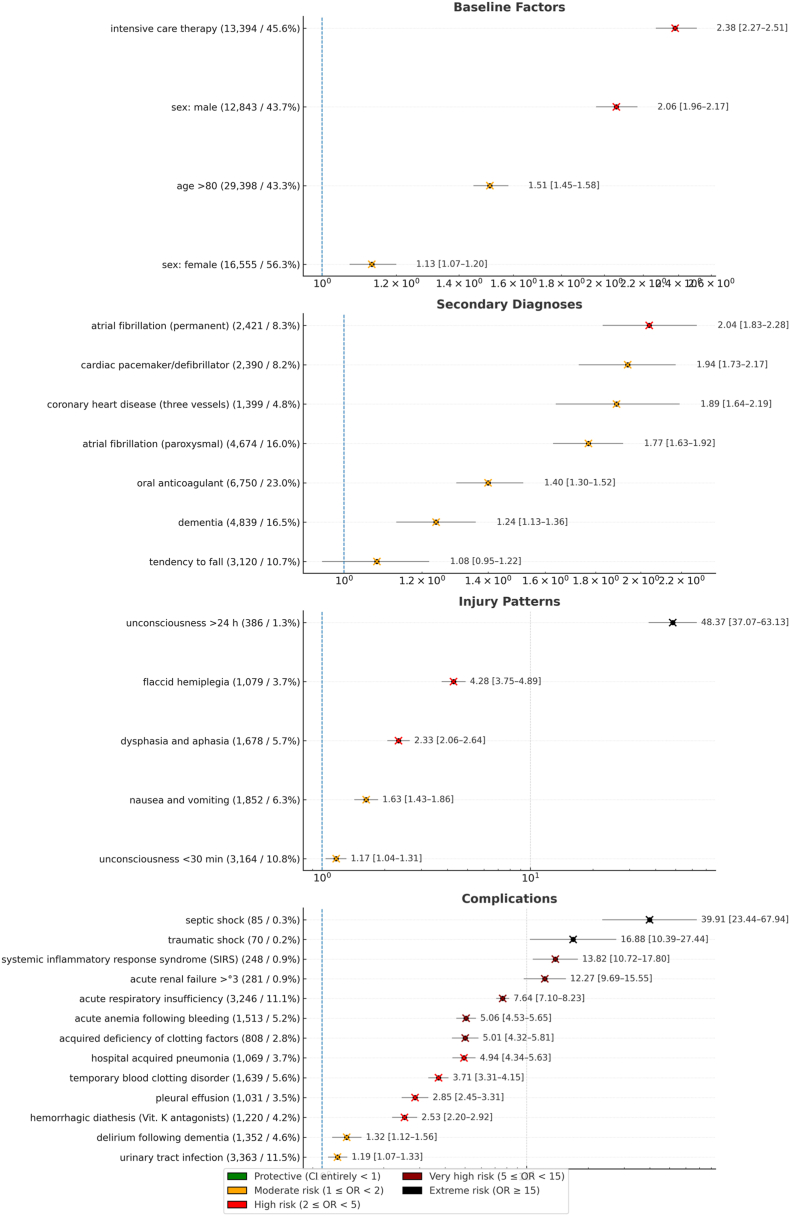
Forrest Plot of the key findings including absolute and relative frequencies and providing Odds ratio (OR) and 95% confidence interval (CI) for in-hospital mortality of the 29,398 included geriatric patients above 80 years with traumatic subarachnoid hemorrhage (tSAH).

Acute renal failure >°3 (155 deaths/281 diagnoses, OR 12.27) was strongly associated with mortality. Haematological complications, including acute anemia (509 deaths/1513 diagnoses, OR 5.06) and acquired deficiency of clotting factors (270 deaths/808 diagnoses, OR 5.01), were also associated with increased risk (Fig. 4).

## 4 Discussion

In this nationwide cohort of 67,684 patients with primary diagnosis of tSAH in Germany, we demonstrate a high overall incidence (34.4 per 100,000) with a marked increase in patients aged ≥80 years. We proved a substantial in-hospital mortality of 9.1%, particularly in the elderly, and found strong associations between mortality and cardiovascular comorbidities as well as systemic complications. Compared to other conditions, this rate is lower than that reported for cervical spinal cord injuries (11.7%) but higher than for periprosthetic joint infections (3.5%) (Schindler et al., 2025; Reinhard et al., 2024).

The observed age gradient with almost quadrupled incidence in patients above 80 years is in line with prior studies on TBI and intracranial hemorrhage, which identify advanced age as a major determinant of poor outcome (Turkstra et al., 2020). Similarly, the higher mortality in male patients aligns with existing literature (Steudel et al., 2005). Although the underlying mechanisms remain incompletely understood.

Cardiovascular comorbidities, including permanent atrial fibrillation, oral anticoagulant use, presence of a pacemaker/defibrillator, and advanced coronary artery disease, were among the variables most strongly associated with in-hospital mortality. These findings are consistent with previous studies demonstrating the impact of comorbidity burden and anticoagulation on outcomes after intracranial hemorrhage (Xiong et al., 2019; Lim et al., 2021; Powers et al., 2020). Our data extend these observations to a large, nationwide cohort of tSAH patients.

Markers of injury severity, such as prolonged unconsciousness (>24 h), flaccid hemiplegia, cerebral edema, and skull fractures, were also strongly associated with mortality. These findings are in line with existing literature stating cerebral edema as predictor for poor outcome after traumatic brain injury (Cook et al., 2020). However, these variables likely reflect the extent of primary brain injury and overall disease severity rather than representing independent effects. This is supported by another study, which found concomitant injuries with abbreviated injury scale ≥2 to be associated with increased in-hospital mortality (Tiruneh et al., 2020). The same can be interpreted for intensive care unit treatment and mortality, as intensive care unit admission likely reflects higher initial severity and complication burden rather than being a causal factor.

Systemic complications showed the strongest associations with mortality, particularly septic shock, traumatic shock, renal failure, and respiratory complications. These findings highlight the importance of secondary systemic deterioration in tSAH. From a clinical perspective, this underscores the need for early recognition and aggressive management of complications, especially respiratory failure and sepsis as a traumatized brain is extremely vulnerable to hypoxia (Bratton and Davis, 1997; Li et al., 2022). Although clinically plausible, our nationwide data are the first to demonstrate the profound impact of systemic complications on in-hospital mortality after tSAH, underscoring the need for their early recognition and management. These findings inform risk stratification but do not establish treatment effects. However, preventive strategies, such as standardized protocols to reduce hospital-acquired pneumonia, may be particularly relevant in high-risk populations.

The effect of the alcohol blood concentration in terms of mortality after TBI remains a topic of discussion with controversial reports (Powers et al., 2020; Tien et al., 2006; Salim et al., 2009; Berry et al., 2010). While our data show an association with lower in-hospital mortality, this finding must be interpreted cautiously. Due to the lack of detailed clinical severity measures, residual confounding cannot be excluded, and no causal or protective effect can be inferred. However, this likely reflects confounding as younger age or less severe injuries.

In patients aged ≥80 years, associations were generally more pronounced, particularly for cardiovascular comorbidities and respiratory and septic complications. This highlights the increased vulnerability of geriatric patients, likely reflecting frailty, multimorbidity, and reduced physiological reserve. In sum, tSAH patients with comorbid cardiovascular disease (especially those with atrial fibrillation on anticoagulants) or (acute) renal dysfunction are at elevated risk of in-hospital death, underscoring the need for aggressive monitoring and possibly adjusted treatment algorithms.

Overall, this large nationwide dataset provides robust epidemiological insights and highlights clinically relevant patterns of association. However, given the lack of detailed clinical data, the findings primarily contribute to risk stratification at a population level rather than mechanistic understanding.

### 4.1 Limitation and strengths

This study has several important limitations. First, due to its observational design, compromising anonymized data from the InEK database and the absence of multivariable modelling, only associations can be inferred, and causality cannot be established. The results are likely subject to confounding, particularly by age, frailty, and overall disease severity. In addition, the lack of multivariable adjustment precludes assessment of independent effects and may substantially overestimate the strength of observed associations.

Second, important clinical variables were not available, including Glasgow Coma Scale scores, radiological severity measures, differentiation of injury subtypes, and cause of death. This limits both risk stratification and mechanistic interpretation. Moreover, several variables identified as strongly associated with mortality (e.g., prolonged unconsciousness, septic shock) likely represent proxies of disease severity or terminal events rather than independent risk factors.

Third, the outcome measure was restricted to in-hospital mortality. Deaths occurring before hospital admission or after discharge were not captured. Thus, the reported mortality rates likely underestimate the true disease burden and do not reflect long-term outcomes.

Finally, data were only available from 2019 onward, overlapping with the SARS-CoV-2 pandemic. Changes in healthcare delivery, hospital pathways, and ICU capacity during this period may have influenced both incidence and outcomes, although this could not be assessed.

Despite these limitations, the study has several key strengths. It represents a nationwide, near-complete cohort of hospitalized tSAH patients in Germany with a very large sample size. The administrative dataset provides comprehensive information on comorbidities and in-hospital complications. In addition, the dedicated analysis of patients aged ≥80 years offers important insights into a rapidly growing and particularly vulnerable population.

## 5 Conclusion

This nationwide analysis demonstrates that advanced age, cardiovascular comorbidities, severe neurological impairment, and systemic complications are strongly associated with in-hospital mortality in patients with tSAH. These findings should be interpreted as associations rather than causal relationships.

Improved identification of high-risk patients, particularly elderly individuals with significant comorbidity burden, may support targeted monitoring and early intervention strategies. Future studies incorporating detailed clinical data and multivariable modelling are needed to better define independent risk factors and inform optimized treatment pathways.

## Informed consent statement

The study was carried out in accordance with the ethical standards of the Declaration of Helsinki of 1975. The data was acquired anonymized. According to national regulations, formal ethics approval was not required for analyses of anonymized administrative data.

## Author contributions

All authors contributed to the interpretation of data and were involved in drafting the manuscript. JR, MS, JS, JK, AH, MR, NOS, VA, TR, SL made substantial contributions to the conception and design of the study. JR, AH, SL participated in the acquisition of data, analysis, and statistics. All authors read and approved the final manuscript.

## Data availability statement

On request, data is available at the authors’ institution.

## Funding statement

This research did not receive any specific grant from funding agencies in the public, commercial, or not-for-profit sectors.

## Declaration of competing interest

The authors declare that they have no known competing financial interests or personal relationships that could have appeared to influence the work reported in this paper.

## References

[bib1] Berry C., Salim A., Alban R. (2010). Serum ethanol levels in patients with moderate to severe traumatic brain injury influence outcomes: a surprising finding. Am. Surg..

[bib2] Bratton S.L., Davis R.L. (1997). Acute lung injury in isolated traumatic brain injury. Neurosurgery.

[bib3] Cook A.M., Morgan Jones G., Hawryluk G.W.J. (2020). Guidelines for the acute treatment of cerebral edema in neurocritical care patients. Neurocritical Care.

[bib4] Dewan M.C., Rattani A., Gupta S. (2019). Estimating the global incidence of traumatic brain injury. J. Neurosurg..

[bib5] Griswold D.P., Fernandez L., Rubiano A.M. (2022). Traumatic subarachnoid hemorrhage: a scoping review. J. Neurotrauma.

[bib6] Guan B., Anderson D.B., Chen L. (2023). Global, regional and national burden of traumatic brain injury and spinal cord injury, 1990-2019: a systematic analysis for the global burden of disease study 2019. BMJ Open.

[bib7] León-Carrión J., Domínguez-Morales Mdel R., Barroso y Martín J.M. (2005). Epidemiology of traumatic brain injury and subarachnoid hemorrhage. Pituitary.

[bib8] Li M., Wang R., Fang Q.X. (2022). Association between Traumatic Subarachnoid Hemorrhage and acute respiratory failure in moderate-to-severe traumatic brain injury patients. J. Clin. Med..

[bib9] Lim X.T., Ang E., Lee Z.X. (2021). Prognostic significance of preinjury anticoagulation in patients with traumatic brain injury: a systematic review and meta-analysis. J. Trauma Acute Care Surg..

[bib10] Long B., Koyfman A., Runyon M.S. (2017). Subarachnoid hemorrhage: updates in diagnosis and management. Emerg. Med. Clin..

[bib11] Powers A.Y., Pinto M.B., Tang O.Y. (2020). Predicting mortality in traumatic intracranial hemorrhage. J. Neurosurg..

[bib12] Reinhard J., Lang S., Walter N. (2024). In-hospital mortality of patients with periprosthetic joint infection. Bone Jt Open.

[bib13] Rhodes H.X., Locklear T., Pepe A. (2023). Can pre-hospital medical management predict In-Hospital mortality in trauma?. Am. Surg..

[bib14] Salim A., Teixeira P., Ley E.J. (2009). Serum ethanol levels: predictor of survival after severe traumatic brain injury. J. Trauma.

[bib15] Schindler M., Krückel J., Straub J. (2025). Risk factors for In-Hospital mortality in cervical spinal cord injuries: a nationwide, cross-sectional analysis of concomitant injuries, comorbidities, and treatment strategies in 3.847 cases. Spine J..

[bib16] Schwenkreis P., Gonschorek A., Berg F. (2021). Prospective observational cohort study on epidemiology, treatment and outcome of patients with traumatic brain injury (TBI) in German BG hospitals. BMJ Open.

[bib17] Steudel W.I., Cortbus F., Schwerdtfeger K. (2005). Epidemiology and prevention of fatal head injuries in Germany--trends and the impact of the reunification. Acta Neurochir (Wien).

[bib18] Steyerberg E.W., Wiegers E., Sewalt C. (2019). Case-mix, care pathways, and outcomes in patients with traumatic brain injury in CENTER-TBI: a European prospective, multicentre, longitudinal, cohort study. Lancet Neurol..

[bib19] Tien H.C., Tremblay L.N., Rizoli S.B. (2006). Association between alcohol and mortality in patients with severe traumatic head injury. Arch. Surg..

[bib20] Tiruneh A., Siman-Tov M., Givon A. (2020). Comparison between traumatic brain injury with and without concomitant injuries: an analysis based on a national trauma registry 2008-2016. Brain Inj..

[bib21] Tropeano M.P., Spaggiari R., Ileyassoff H. (2019). A comparison of publication to TBI burden ratio of low- and middle-income countries versus high-income countries: how can we improve worldwide care of TBI?. Neurosurg. Focus.

[bib22] Turkstra L.S., Mutlu B., Ryan C.W. (2020). Sex and gender differences in emotion recognition and theory of mind after TBI: a narrative review and directions for future research. Front. Neurol..

[bib23] Xiong C., Hanafy S., Chan V. (2019). Comorbidity in adults with traumatic brain injury and all-cause mortality: a systematic review. BMJ Open.

[bib24] Younsi A., Unterberg A., Marzi I. (2023). Development and first results of a national databank on care and treatment outcome after traumatic brain injury. Eur. J. Trauma Emerg. Surg..

